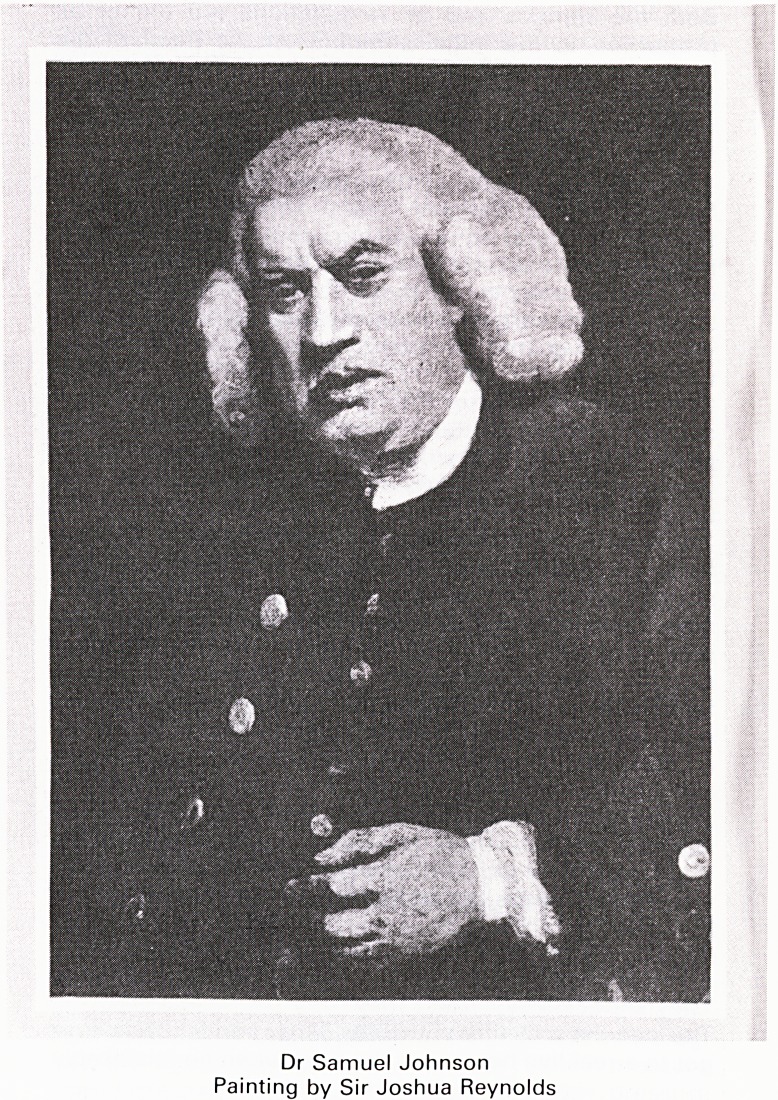# Dr Robert Levet: An Obscure Practiser in Physic

**Published:** 1986-04

**Authors:** John Griffiths


### Dr. Robert Levet: an obscure practiser in physic

**John Griffiths**

Robert Levet (or Levett: the name is spelt variously) would have passed unnoticed into the mists of antiquity had he not in 1746 met Dr. Samuel Johnson.

He was born in Hull in or about 1701: his origin was obscure: as a young man he went first to London as a servant and then to Paris, being employed there as a waiter in a restaurant which was patronised by members of the medical profession. He showed a lively interest in their work and must have impressed them not only with his intelligence and enthusiasm, but with a natural aptitude for their profession: they gave him informal instruction in medicine, supplemented no doubt by what he overheard during his duties in the restaurant: and he got some formal training in pharmacy and anatomy, the cost of which was met by contributions made by some of the doctors to whom he served their meals. He eventually considered that he was well enough equipped to practise medicine himself, and returned to London, gaining a small reputation as a surgeon. He was unlicensed (Boswell makes a derisive reference to the habit of Johnson's servant of calling him 'Dr.' Levet) and wholly without influence or formal qualifications, so that his practice lay among the 'lower people'. His remuneration was pitifully small: he appears to have accepted what his patients could spare from their own penury, and it sometimes took the form of provisions, both solid and liquid. He had a strong sense of economy and though he was the least avaricious of men and never pressed for his dues, he would not waste those given to him in kind: with the result that as he was not infrequently paid with drink, he would consume more than was good for him rather than decline or throw it away. According to Johnson, Levet was 'perhaps the only man who ever became intoxicated through prudence': and 'had all his patients maliciously combined to reward him with meat and strong liquors instead of money he would either have burst like the dragon in the Apocrypha, or been scorched up like Portia by swallowing fire'.

Dr Samuel Johnson
Painting by Sir Joshua Reynolds

Levet went to live in Johnson's house at 17 Gough Square in 1752 and so became one of a motley collection comprising Johnson's negro servant Francis Barber and his wife: Mrs. Williams, a blind and cantankerous minor bluestocking who had some published writing to her credit; Mrs. Desmoulins the daughter of Johnson's godfather; and Poll Carmichael, 'a Scotch Wench, who has her case as a pauper depending in some of the Law Courts.' Poll's 'case as a pauper' must have resembled Jarndyce v. Jarndyce, as was then only too common: she seems to have lived in this odd community for a considerable time, doing duty as cook and kitchenmaid. In a letter to Mrs. Thrale, Johnson thus describes the communal relationships: 'Williams hates everybody: Levett hates Desmoulins and does not love Williams: Desmoulins hates them both: Poll loves none of them'. Luckily they were all devoted to Johnson.

Johnson was a lifelong hypochondriac, and took a morbid interest in his numerous ailments: he had been touched in childhood by Queen Anne for the 'Kings Evil' or scrofula, which had left his hearing and eyesight permanently impaired: and Levet seems to have acted as his resident physician, under the general direction of Dr. Lawrence, a medical man of high standing in his profession. In spite of the fact that Johnson once described Levet as 'a brutal fellow', he added 'but I have a good regard for him, for his brutality is in his manners, not in his mind': and he plainly derived substantial support from his companionship. Johnson, too, had a warm hearted compassion for the poor and unfortunate: when, in a conversation with Oliver Goldsmith, Boswell mentioned Levet, Goldsmith observed 'he is poor and honest, which is recommendation enough to Johnson'. Levet and Johnson shared a late breakfast daily, in silence: Levet made the tea: after which Levet would depart on his 'walk' or round of visits 'from Houndsditch to Marylebone' returning to 17 Gough Square often late at night. Johnson had a high opinion of Levet's medical skills: he said that 'he should not be satisfied though attended by all the College of Physicians unless he had Mr. Levett with him'.

When he was about 60 years of age, Levet formed an association with a 'street walker' whom he used to meet in a coal cellar in Fetter Lane. This lady persuaded him that she was entitled to a large fortune, of which she was unjustly deprived, and she got the impression that he was a distinguished physician with a large and remunerative practice. On this basis of mutual misunderstanding or deception they married, and Levet appears to have left his room in Johnson's house and taken his bride elsewhere. The sound of the wedding bells had hardly died away when Levet was arrested for some debts of his wife. Johnson rescued him from her creditors, and the couple separated. Shortly afterwards Mrs. Levet was tried at the Old Bailey for picking pockets, and Levet attended the trial in the hope of hearing his wife sentenced to be hanged. She conducted her own defence, and was acquitted, so Levet had to rely on less drastic means for ridding himself of this burden. The marriage was in due course dissolved, and Levet resumed his residence with Johnson.

Levet was instrumental in introducing Johnson to Bennet Langton, whom Levet had met in a house which he visited in his professional capacity. Langton came of a very ancient and distinguished family and was a scholarly and charming man: he became a lifelong friend of Johnson. Levet was also instrumental in provoking one of Johnson's most magisterial and well known reproofs: being irritated by hearing a gentleman ask Levet a variety of questions concerning him when he was sitting by, Johnson exclaimed 'Sir, you have but two topicks, yourself and me: I am sick of both'.

In 1785, three years after his death, the Gentlemans Magazine published an 'account' of Levet: 'His person was middle sized and thin: his visage swarthy, adust, and corrugated. His conversation, except on professional subjects barren. When in dishabille, he might have been mistaken for an alchemist, whose complexion had been hurt by the fumes of the crucible, and whose clothes had suffered from the sparks of the furnace.'

Robert Levet, whom James Boswell introduces to posterity as 'an obscure practiser of physic among the lower people', was, with all his oddities, an unassuming, undemanding, and dedicated medical man who endeared himself without effort to one of the most formidable intellects of his or any age, and to innumerable patients living in conditions of squalor, ignorance, filth and poverty which defy the liveliest imagination. Although an autodidact, he must have had considerable professional skill, which he maintained and improved by regular attendance at the lectures of the great John Hunter. He died suddenly in bed, in his room in Johnson's house on the 17th of January 1782. In the following March, in a letter to Bennet Langton, Johnson writes 'my dear old friend Mr. Levett, to whom as he used to tell me, I owe your acquaintance died a few weeks ago suddenly in his bed: there passed not, I believe, a minute between health and death...I thought with uncommon earnestness that however I might alter my mode of life or withersoever I might remove I would endeavour to retain Levett about me...'

Practical as ever, Johnson sought Levet's relations by advertisement and distributed his scanty effects among those whom he discovered by this means.

Johnson was deeply grieved by Levet's death, which drew from him nine moving stanzas of lyric verse: two of these stanzas enshrine at once their subject's quality and the poet's gift:

No summons mocked by chill delay
No petty gain disdained by pride
The modest wants of every day
The toil of every day supplied

His virtues walked their narrow round
Nor made a pause nor left a void
And sure th' Eternal Master found
The single talent well employed.

## Figures and Tables

**Figure f1:**